# Evaluation of Process Parameters for Continuous Manufacturing of Quetiapine Fumarate Immediate Release Tablets Using Twin Screw Wet Granulation

**DOI:** 10.1007/s11095-025-03859-7

**Published:** 2025-04-14

**Authors:** Tejaswini Naguboyina, Preethi Lakkala, Siva Ram Munnangi, Sateesh Kumar Vemula, Michael Repka

**Affiliations:** 1 Department of Pharmaceutics and Drug Delivery, School of Pharmacy, The University of Mississippi, University, MS 38677 USA; 2 Pii Centre for Pharmaceutical Technology, The University of Mississippi, University, MS 38677 USA; 3 Department of Pharmaceutics, School of Pharmaceutical Sciences, Lovely Professional University, Phagwara, Punjab India

**Keywords:** agglomeration, granules, liquid-to-solid ratio, process parameters, twin-screw wet granulation

## Abstract

**Purpose:**

Granulation is one of the important unit operations in the manufacturing of solid dosage forms like tablets and capsules that regulate the quality of end products. It is a process of particle enlargement by agglomeration technique, which improves flow properties, compressibility, reduction of dust formation, drug content uniformity, dissolution rates, and overall product stability. Traditionally, it has been a batch process due to a better understanding of the process. However, there has been a shift towards continuous manufacturing using a Twin-screw granulator, which is more robust, scalable, and versatile for a wide range of applications.

**Methods:**

This work presents the innovative use of Twin screw wet granulation (TSWG) in the development of Quetiapine fumarate (QTF) immediate release tablets. Various process parameters (Screw configuration, liquid-to-solid (L/S) ratios), and binders (HPC and PVP) were evaluated to determine their effect on granule quality. Further, the obtained granules were tested for particle size distribution and flow properties.

**Results:**

A higher percentage of uniform-sized granules were yielded with three mixing zones even with a lower liquid addition compared to that of one mixing zone with a higher liquid addition. These granules were further tableted and tested for their hardness, friability, disintegration, and dissolution. The tablets disintegrated and released the drug (~ 95%) rapidly within 5 min in 0.1 N HCl due to QTF’s high solubility and porosity of granules.

**Conclusions:**

Overall, the understanding of process parameters and their influence on granule and tablet characteristics would help establish a more robust and continuous manufacturing of dosage forms.

**Graphical Abstract:**

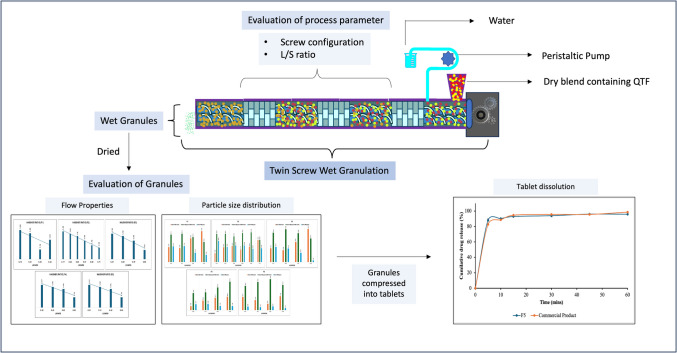

## Introduction

In the realm of pharmaceutical manufacturing of solid dosage forms, attaining optimum drug delivery and uniformity is of utmost importance to ensure the safety and efficacy of medications [1]. While direct compression is a simple and cost-effective technique for producing solid oral dosage forms such as tablets, it can be hindered by the segregation, poor flow properties, poor compressibility, etc. and it is often not suitable for a highly potent active pharmaceutical ingredient (API). Additionally, static charges generated during dry mixing can cause clumping of API and/or excipients, leading to poor content uniformity [2]. To overcome these issues, granulation techniques are often used. Granulation converts fine or coarse powders into uniform granules, playing a critical role in tablet and capsule production, influencing product effectiveness, handling, and quality [3]. It increases density, minimizes dust formation, and enhances flow properties, facilitating smoother processing during blending, filling, and compression phases. Factors such as particle size, binders, granulator type, and drying conditions affect granule characteristics [4].

Granulation methods, classified as dry or wet, are chosen based on drug stability. Dry granulation uses high-pressure compression (e.g., slugging or roller compaction) to form granules without liquid, while wet granulation incorporates a liquid binder to improve flow, compressibility, and uniformity, especially for heat-sensitive APIs [5]. Wet granulation methods include fluid bed and high-shear techniques, though they require multiple equipment, larger material quantities, and controlled environments, making them time-intensive [6, 7]. The wet granulation process comprises wetting and nucleation, coalescence and consolidation, and attrition and breakage. Wetting distributes liquid to form cohesive bridges, nucleating granules, while coalescence consolidates and strengthens them [8]. Attrition refines granules, affecting particle size distribution, porosity, and uniformity, which are critical for pharmaceutical applications [9, 10].

**Fig. 1:**
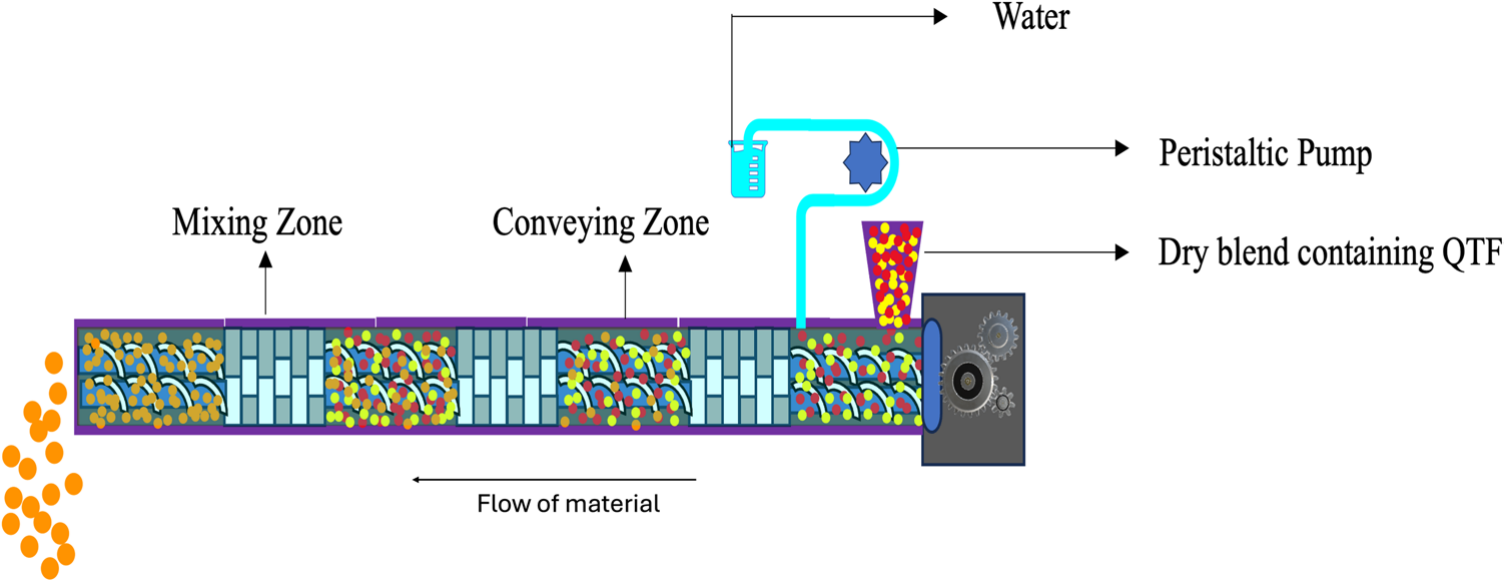
Schematic diagram of twin screw wet granulation process [13].

TSWG is a novel continuous process with several benefits over batch granulation methods. TSWG enables the creation of granules with lower liquid amounts and improved consistency compared to high-shear batch mixers [11]. Because it is continuous, it may be used for both high and low-volume manufacturing and provides more flexibility for in-line process control methods. In TSWG, the incorporation of liquid or solvent is crucial for binding and agglomerating powder particles into granules. A peristaltic pump is typically used to introduce the liquid binder into the system through a liquid injection vent, preferably positioned near the feeding zone (Fig. 1). Depending on the quantity of binder required it can be incorporated into either the dry blend or directly into the liquid phase. If the quantity of binder is minimal, it is preferable to introduce it directly into the liquid phase to ensure proper dispersion and homogeneity. This approach helps optimize granulation efficiency and ensures uniform distribution of the binder throughout the mixture, resulting in consistent granule properties. A recent study by Kolipaka *et al*. (2024) investigated the application of non-volatile solvents as granulating binders in TSWG, highlighting its potential for further process enhancements [12].

TSWG is influenced by solid feed rate, feed RPM, screw configuration, screw RPM, and liquid-to-solid (L/S) ratio. Based on the flowability of the physical mixture, various types of feeders are available to supply the physical mixture into the barrel inlet, such as screw feeders, gravity feeders, and vibratory feeders [13]. The configuration of screws is determined by the specific characteristics of the material being processed and the manufacturing needs. The components on the screw shaft can be interchanged, enabling the creation of a tailored screw configuration to fulfil the unique demands of specific required processes [14]. Conveying elements, kneading, or mixing elements, and distributive elements are different types of screw elements typically used in TSWG [15]. Conveying elements play a crucial role in facilitating the movement of materials between mixing zones by reducing mechanical energy and shear force. Their main purpose is to transport materials within mixing systems effectively. Kneading blocks are comprised of individual kneading discs and function as mixing zones within TSG processes. These blocks apply substantial mechanical energy to the moistened powder mass, resulting in the generation of high shear forces, compaction, and thorough mixing [16]. The kneading discs within a kneading block are commonly positioned at varying angles such as 30°, 60°, or 90°. The number of kneading elements plays a significant role in determining the characteristics of the granules produced [17]. Additionally, there is a correlation between the kneading elements and the torque exerted during the process. Lowering the screw speeds with a constant feed rate can cause high fill levels, leading to material compaction and potential blockages at high mass loads. Conversely, increasing the screw speeds at a constant feed rate can lead to low barrel fill levels, potentially starving the screw channels of powder, resulting in limited compaction and particle interaction [18]. Variations in fill level can significantly impact binder distribution and granule properties, making screw speed a crucial consideration during the scale-up of twin-screw granulation. Optimizing the L/S ratio is essential for wet granulation procedures since it has a direct impact on the characteristics and quality of the final granules [13]. The quantity of the solid blend to be granulated determines how much liquid or solvent is needed, and the L/S ratio needs to be carefully adjusted based on several variables, including the binder's properties. For example, to properly disperse and wet the powder particles, high-viscosity binders might need more solvent to ensure efficient binding and granule formation. However too much liquid can cause over-wetting, which can cause an excessive amount of agglomeration [19]. Recent advancements in TSWG have aimed to eliminate the drying step by incorporating in-process drying techniques. Haser *et al*. developed an innovative in-barrel drying method that utilizes a vacuum system to dry granules within the extruder, thereby removing the need for an additional external drying step [20].

The aim of this study was to investigate the effects of different binders and process parameters utilized in TSWG on granule properties. Specifically, this innovative study examined how variations in screw configuration, binder concentration, liquid feed rate, and solid feed rate influence key granule characteristics such as size distribution and flowability. Quetiapine Fumarate (QTF) was chosen as the model drug for this research. By systematically varying these parameters, the study aimed to identify optimal conditions for producing granules with desirable properties for subsequent tablet manufacturing. The findings are expected to provide insights into the formulation strategies and process optimization for achieving consistent and high-quality granule production in solid oral dosage forms.

## Materials and Methods

### Materials

QTF was obtained from RIA International LLC; Klucel EXF (Hydroxypropyl cellulose) was obtained from Ashland; Kollidon 30 (Polyvinylpyrrolidone K30) was obtained from BASF; Cab-o-sil 5-MP (Fumed Silica) was obtained from Cabot Corporation; Ac-Di-sol (Sodium Starch Glycolate) was obtained from IFF’s pharma solution; Avicel pH 101 (Microcrystalline Cellulse) was obtained from Sigma-Aldrich; Parteck M 200 (Mannitol) was procured from Merck; Hydrochloric acid (HCl) and other chemicals were obtained from Fisher Scientific.

### Methods

#### UV–Visible Spectroscopy

A calibration curve for QTF was plotted by using water as a solvent using a GENESYS™ 180 UV–Visible Spectrophotometer (Thermo Fisher Scientific, Waltham, MA, USA) at a λ_max_ of 290 nm. Precisely 10 mg of QTF was transferred to 20 mL of water to form 0.5 mg/mL stock solution from which further dilutions of 0.012 mg/mL, 0.025 mg/mL, 0.037 mg/mL, 0.050 mg/mL,0.075 mg/mL were prepared [21].

#### Processing of QTF-Loaded Granules

##### Preparation of the Dry Blend

Five batches (F1 to F5) with a batch size of 50 g were prepared according to the composition provided in Table I. The drug load was consistently maintained at 40% w/w for all batches. Microcrystalline cellulose (MCC) and mannitol were used as diluents, while hydroxypropyl cellulose (HPC) and Polyvinylpyrrolidone K30 (PVP) were tested as binders at varying concentrations. Croscarmellose sodium (CCS) was used as a disintegrant, and fumed silica was incorporated as a glidant in the formulations. Initially, a pre-blend was prepared by mixing QTF with various polymers and excipients, excluding fumed silica. All the pre-blend materials were sifted through an ASTM 30 sieve, and the sifted materials were blended using a V-blender at 25 rpm for 10 min. Subsequently, fumed silica was weighed separately, sifted through an ASTM 30 sieve, and then added to the pre-blend, which was blended in the V-blender for 5 min at 25 rpm.

**Table I:** Formulation Composition of Different Formulations (F1 to F5).

Formulation	API (%)	MCC (%)	Mannitol (%)	HPC (%)	PVP (%)	CCS (%)	Fumed Silica (%)
F1*	46.1	23.0	23.0	5.0	-	2.5	0.5
F2*	46.1	24.0	24.0	-	3.0	2.5	0.5
F3*	46.1	23.0	23.0	-	5.0	2.5	0.5
F4**	46.1	23.0	23.0	-	5.0	2.5	0.5
F5***	46.1	23.0	23.0	-	5.0	2.5	0.5

*Single mixing zone; ** Two mixing zones; *** Three mixing zones

##### Wet Granulation Using Twin Screw Granulator

The physical mixtures were granulated using a Process- 11 (11 mm) Twin-Screw Extruder (Thermo Fisher™, Waltham, MA). Water was introduced through a peristaltic pump via a liquid injection port at zone 1. The unit dose of QTF for each tablet was set at 460.55 mg (Eq. 400 mg base). Various L/S ratios were evaluated for each batch to determine the optimum values. Formulations F1, F2, and F3 were processed using a single mixing zone (Zone 2) with 5% HPC as a binder in F1, 3% and 5% PVP in F2 and F3, respectively. Formulations F4 and F5 contained 5% PVP and were processed with two mixing zones (Zones 2 and 4) and three mixing zones (Zones 2, 4, and 6), respectively. Each mixing zone consists of 8 elements, each element has a width of 0.25 mm. The elements are arranged with a 60-degree offset angle between them. Additionally, star-shaped comb mixer elements were incorporated near the discharge section to break down the large lumps and large granules into smaller granules. For all formulations, the screw speed and feed rates were kept constant at 50 RPM and 2 gm/min respectively, and the temperature for all zones was set at 18°C. Different L/S ratios for F1 to F5 were evaluated, as shown in Table II, and the torque was maintained below 50% for all formulations. After granulation, the extruded granules were dried in an oven at 40°C for 1–2 h until the moisture content reached 2–5%.

**Table II:** Evaluation of Binders and Process Parameters.

S.No	Formulation	Screw configuration	Binder concentration	L/S ratio
1	F1	Mixing zone in zone 2	5% HPC	0.16
2	F1			0.21
3	F1			0.32
4	F1			0.42
5	F2	Mixing zone in zone 2	3% PVP	0.17
6	F2			0.23
7	F2			0.35
8	F2			0.47
9	F2			0.59
10	F2			0.71
11	F3	Mixing zone in zone 2	5% PVP	0.47
12	F3			0.63
13	F3			0.79
14	F3			0.95
15	F4	Two mixing zones in zones 2 & 4	5% PVP	0.32
16	F4			0.42
17	F4			0.52
18	F4			0.63
19	F5	Three mixing zones in zones 2, 4 & 6	5% PVP	0.21
20	F5			0.31
21	F5			0.42
22	F5			0.52

##### Moisture Analysis

Moisture analysis for all the dried granules was performed using a loss-on-drying method using an infrared moisture balance. Approximately 5 g of each sample was subjected to analysis by exposing it to a temperature of 105°C and the endpoint was determined when the change in weight loss was less than 1 mg for 30 s [22].

#### Evaluation of Granules

##### Particle Size Distribution (PSD)

PSD of all the granules prepared was assessed through sieve analysis utilizing a vibratory sieve shaker (Performer III SS- 3, Gilson Inc., USA). This analysis involved the use of a set of sieves with mesh sizes of #16, 20, 30, 40, 60, 80, and 120. A precisely measured quantity of granules was placed in the sieve set, followed by a 10 min analysis at an amplitude of 6. Subsequently, the weight of granules retained on each sieve was accurately determined, and the weight fraction was calculated using the below formula [23].

$$Material\;retained\;on\;sieve\;\left(\%\right)=\frac{Material\;retained\;on\;sieve\;\left(g\right)}{Total\;Material\;taken\;for\;test}\times100$$

##### Flow Properties

The flow characteristics of all granules were evaluated by measuring the bulk density (BD), tap density (TD), and Hausner ratio (HR). A 250 mL measuring cylinder was weighed, and its weight was recorded. The granules were accurately weighed and transferred into the measuring cylinder. The mass of the cylinder with the granules and the volume of the untapped granules were recorded. The cylinder was then tapped for 10, 500, and 1250 taps, or until the volume decrease was less than 2 mL, to determine the tap density. If the volume difference exceeded 2 mL, an additional 1250 taps were performed until two consecutive measurements showed a difference of less than 2 mL [24]. The final tapped volume of the granules was recorded, and the densities were calculated using the formulas provided below

$$\begin{array}{l}\mathrm{Bulk}\;\mathrm{density}=\mathrm{Mass}\;\mathrm{of}\;\mathrm{granules}/\mathrm{untapped}\;\mathrm{volume}\\\mathrm{Tapped}\;\mathrm{density}=\mathrm{Mass}\;\mathrm{of}\;\mathrm{granules}/\mathrm{tapped}\;\mathrm{volume}\\\mathrm{Hausner}'\mathrm s\;\mathrm{ratio}=\mathrm{Tapped}\;\mathrm{density}/\mathrm{Bulk}\;\mathrm{density}\end{array}$$

#### Sizing and Milling of Granules

The granules were passed through an ASTM mesh size 30. The granules retained on the mesh were then transferred to a bench-top Fitz mill and gently milled enough to pass through 30 mesh to achieve uniform size. The granules obtained were sifted through mesh size 30 multiple times until all granules were of uniform size.

#### Fourier Transform Infrared (FTIR) Spectroscopy

FTIR was performed to know whether there were any chemical interactions between the materials within the formulation. An infrared spectrophotometer (Cary 630 FTIR, Agilent Technologies, USA) was used at room temperature (25°C). The IR transmission spectra were recorded between 650 and 4000 cm^−1^, with a resolution of 8 cm^−1^. An average of 32 repeat scans were taken to obtain each spectrum. Pure API, polymers, other tableting excipients, physical mixtures, and granules were checked for interactions using the spectrophotometer [25].

#### Tableting

Magnesium stearate (1%) was added extra granularly to the milled granules and was blended in a V-blender at 25 rpm for 10 min. These lubricated granules were then compressed into tablets on a Natoli NP-RD10 A single-punch tablet press using an oval shaped punch (0.4331 * 0.7402 inches) with a targeted hardness of 7–10 kP.

#### Tablet Characterisation

The assay (*n* = 3) was conducted on the compressed tablets. For each sample, 10 tablets were crushed and triturated using a mortar and pestle. An amount of the powder equivalent to the weight of one tablet was collected and dissolved in 1000 mL of water, followed by UV analysis.

The average weight of the tablet was measured by taking a sample of 10 tablets. The thickness of ten tablets was measured using digital vernier callipers to assess uniformity. The hardness of the tablets was evaluated by testing ten units with a hardness tester (VK 200, Optimal) to determine the average tablet hardness. For friability testing, tablets (*n* = 10, as each unit weighed more than 650 mg) were initially weighed (W) and then subjected to testing using a friability tester (FT2, Sotax) at a speed of 25 rpm for 4 min [26]. After testing, the tablets were reweighed (W_0_), and the percentage weight loss was calculated to determine the tablet friability using the formula provided below.

$$\text{Friability }(\text{\%})=\frac{\text{W}-{\text{W}}_{0}}{\text{W}}*100$$

##### Disintegration

The disintegration time of six tablets (*n* = 6) was assessed using a standard disintegration apparatus (DT2-IS, Dr.Schleuniger Pharmatron) to ensure the consistency and quality of the formulation. The test was conducted in distilled water with the use of discs, maintained at a controlled temperature of 37 ± 0.5°C.

##### *In-vitro* Dissolution Study

Drug release studies were performed on tablets containing 460.55 mg of QTF (Eq. 400 mg base) using a USP Type 2 dissolution apparatus. The dissolution medium consisted of 500 mL of 0.1 N HCl, maintained at 37 ± 0.5 C, with a paddle speed of 100 rpm for 1 h [27, 28]. Given the good solubility of QTF across the gastrointestinal pH range, 0.1 N HCl was selected as the dissolution medium to simulate the physiological gastric environment. The 500 mL volume was sufficient to completely solubilize the administered dose of the API. Samples were collected at intervals of 5, 10, 15, 30, 45, and 60 min. At each time point, 2 mL of the dissolution medium was withdrawn through a cannula equipped with a 0.45 μm filter, and the withdrawn volume was replaced with fresh medium. The collected samples were analyzed using a UV spectrophotometer, as described in Sect. 2.2.1. Finally, the dissolution profile is compared with commercial product’s dissolution profile to check the similarity.

## Results and Discussion

### Twin Screw Wet Granulation

TSWG platform serves as a robust technique for wet granulation. The L/S ratio, regardless of the binder used and the number of mixing zones incorporated, has a direct impact on the granulation process. A lower L/S ratio resulted in an under-granulated mass (fines), while an increased L/S ratio gave rise to good granulation i.e., coarse granules [29]. However, the optimum L/S ratio varied depending on the specific binder used and the number of mixing zones incorporated. The study evaluated two binders for wet granulation: HPC, with low wettability, and PVP, with high wettability [30]. The F1 resulted in a mixture of ungranulated powder and loosely packed larger agglomerates, as HPC is a highly viscous binder with low wettability. This could be due to the formation of a viscous layer on the surface of the binder particles/agglomerates, caused by less water and low retention times in the barrel. The diluents might just be sticking to the surface of the binder with low binding efficiency [31]. A similar observation was reported by Vandevivere *et al*., where they noted that HPMC exhibited a high contact angle with mannitol, leading to poor wettability of the mannitol and consequently poor granule quality [32].

**Fig. 2:**
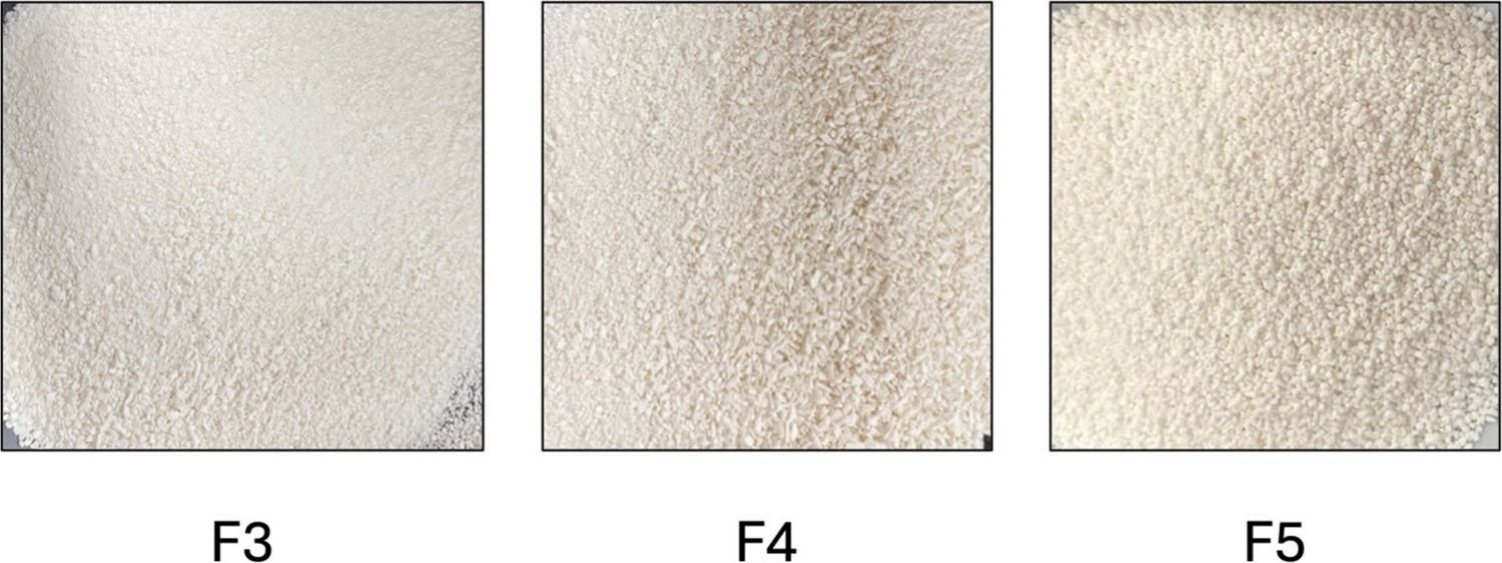
Granules of formulations F3, F4, and F5 at 0.52 L/S ratio.

Further, 3% PVP was included in F2, leading to the formation of compact granules with good strength. However, the presence of ungranulated powder (fines) was also observed, likely due to the low binder concentration [33]. In F3, granules were therefore processed with a higher concentration of PVP (5%), which resulted in good granulation with a minimum volume of fines. To further evaluate the impact of screw configuration (number of mixing zones) on granule quality and liquid uptake, F4 was processed with two mixing zones exerting higher shear compared to that of one mixing zone with the same concentration of binder (5%), resulted in good granulation with lower liquid uptake (lower L/S compared to single mixing zone). Increasing the mixing zones to three in F5 resulted in better granulation with even lower water uptake compared to two and one mixing zones, due to higher shear exerted (Fig. 2). Therefore, as the mixing zones increased, the mixing time and shear also increased, leading to the production of better granules with reduced fines. Water uptake has been reduced from screw configuration having one mixing zone to screw configuration having three mixing zones [29].

### Moisture Analysis

It is crucial that the moisture content in the granules does not exceed 5% to prevent the possibility of microbial growth during storage and also impacts the compaction properties of the granules [34]. On the other hand, if the granules are completely dry, they will break while being compressed into a tablet due to the reduction in compactibility of the granules. To ensure optimal results, the granules were dried until the loss on drying was between 2 and 5%, aligning with the moisture content of the physical mixture [35].

### Characterization of Granules

#### Particle Size Distribution (PSD)

The percentage of optimum granules (those that pass through sieve #20 but are retained in sieve #60), fines (those that pass through sieve #60), and large agglomerates (that are retained in sieve #20) were determined using PSD analysis. To optimize the granulation process and to develop tablets with desired characteristics, it is essential to have a thorough understanding of PSDs. It is recommended to have around 10–20% fines along with granules to fill the voids that may occur during compression [36, 37].

**Fig. 3:**
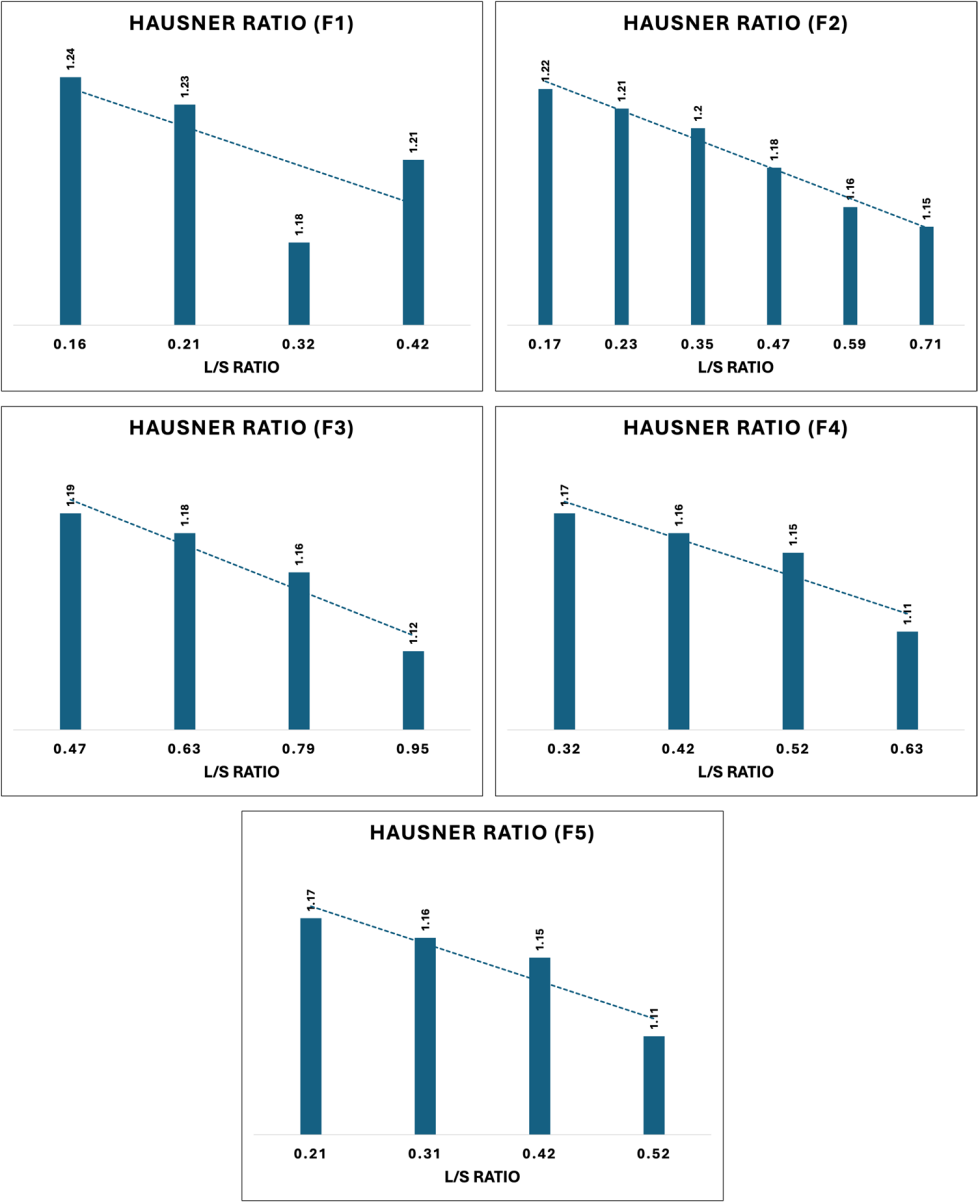
PSDs of granules processed at different L/S ratios (F1 – F5).

The PSDs of formulations F1 through F5 despite the differences in the formulation composition, followed a trend based on the L/S ratios (Fig. 3). As the L/S ratio increased, the percentage of granules with uniform sizes (sieve #20 pass and #60 retain) also increased. Additionally, the higher L/S ratios showed an increase in oversized granules (sieve #20 retain) [38]. On the other hand, there was a decrease in fines (sieve #60 pass) with an increase in L/S ratios. Further, the screw configuration, which exerts high shear with increasing number of mixing zones, was found to affect the percentage of uniform-sized granules produced [39]. Specifically, 75% of the granules were uniform in size when the configuration was F5 (three mixing zones) at an L/S ratio of 0.42.

#### Flow Properties

**Fig. 4:**
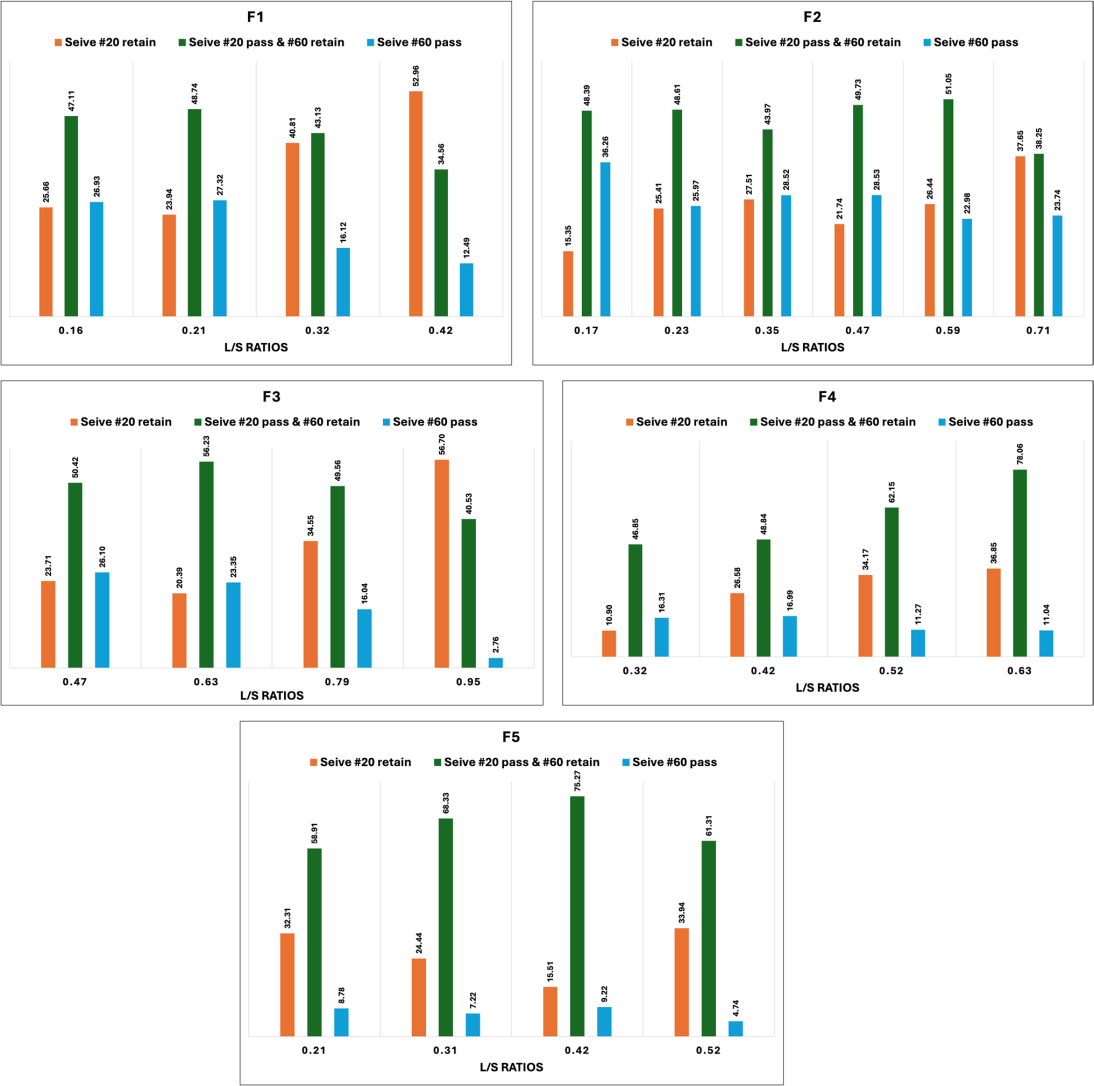
The hausner ratio for formulations processed at different L/S ratios.

To evaluate the flow properties of different granules processed with various binders and screw configurations, their bulk and tap densities were measured to determine the Hausner ratio. All the granules, from F1 through F5, had passable to excellent flow properties, and none of them had poor flow. However, a relationship was observed between the L/S ratios, the binder used, and the number of mixing zones (Fig. 4). Overall, an increase in the L/S ratio resulted in improved flow properties, which can be attributed to the formation of uniformly sized granules, enhanced granule sphericity and a reduced quantity of fines [40, 41]. Formulations containing PVP (F2, F3, F4, and F5) exhibited superior flow properties compared to F1, which utilized HPC. This enhancement is attributed to the excellent wettability of PVP, facilitating uniform distribution throughout the blend and resulting in well-formed granules that improve flow characteristics. Additionally, increasing the binder concentration further enhanced flowability [42]. Additionally, as the number of mixing zones increased from 1 to 3 in Formulations F3 to F5 respectively, flow properties improved. Mixing zones play a crucial role in granule formation by creating an environment that promotes the initial nucleation of granules. They achieve this by bringing powder particles and liquid binder into close contact, followed by facilitating the growth of these nuclei through additional mixing and agglomeration [43].

### FTIR

**Fig. 5:**
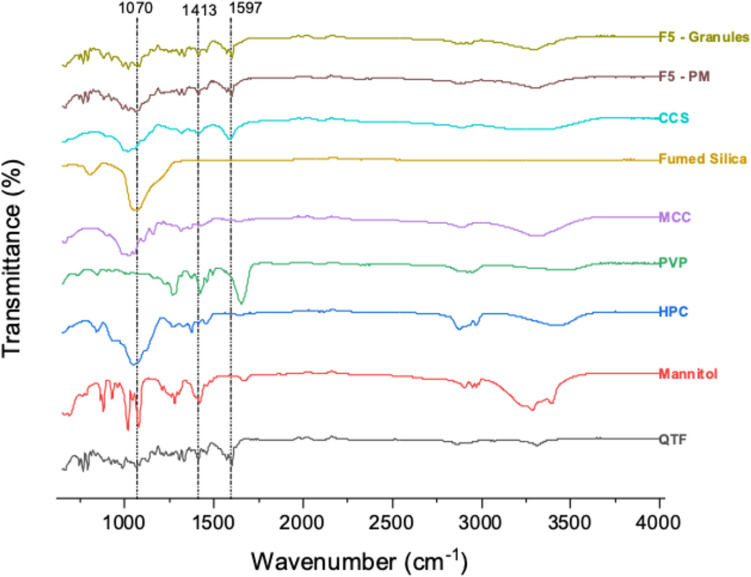
FTIR spectra of QTF, PM, Granules, and excipients.

The FTIR spectra of QTF and other excipients in granules are represented in Fig. 5. The FTIR spectra of QTF exhibited peaks at 3310 (OH vibration), 2941 (C-H bend), 1597 (N–H bending), 1413 (C-H bending), and 1070 (C–C stretching) [1, 44]. The IR spectrum of the physical mixture represents the superimposed spectra of all its constituent materials. In the granules of the lead formulation, no new bonds were formed, no existing bonds were absent, and no significant shifting or broadening of IR bands was observed. This indicates strong compatibility among the components used in the formulation [25].

### Tablet Characterization

The goal was to create QTF immediate release tablets, but achieving this required evaluating various parameters involved in the continuous processing of granules using TSWG. While the formulation aspect remained the same (except for the binder used), changing certain parameters had an impact on the outcome. No single formulation emerged as superior in every aspect, so there is no clear lead formulation. However, F5 processed with three mixing zones at a ratio of 0.52 L/S was chosen for compression into tablets and further characterization, due to the excellent flow properties of granules and uniformly distributed granules. Another reason for selecting F5 was because it was processed with higher mixing zones, which might have resulted in compact granules due to high shear. Studying this formulation could provide insight into whether it is hindering the disintegration and dissolution.

The F5 formulation tablets exhibited an assay value of 96.5%w/w, which is within the acceptable range of 90–110%. The theoretical weight of the tablets is 1000 mg, with an average practical weight of 998.3 ± 1.2 mg. The hardness of the tablets was targeted between 7–10 Kp, and the average measured value was 7.8 ± 1.4 Kp, aligning with the specified limits. The average thickness of the tablets was 8.2 ± 0.05 mm. Additionally, the friability was measured at 0.7%, well below the acceptable threshold of 1%, confirming that the tablets passed the friability test.

### Disintegration

All the tablets (F5) disintegrated in under five minutes, demonstrating rapid disintegration despite the granules being subjected to high shear processing in the twin-screw wet granulator (TSWG). This remarkable disintegration performance can be attributed, in part, to the inclusion of 2.5% disintegrant in the formulation, which likely facilitated the breakdown of the tablet matrix upon contact with the media. Additionally, the inherent porosity of the tablets appears to have played a critical role, enabling the penetration of the disintegration media into the tablet core. This synergistic effect of the disintegrant and porosity underscores the importance of carefully optimized formulation and processing parameters in achieving rapid tablet disintegration.

### Dissolution

**Fig. 6:**
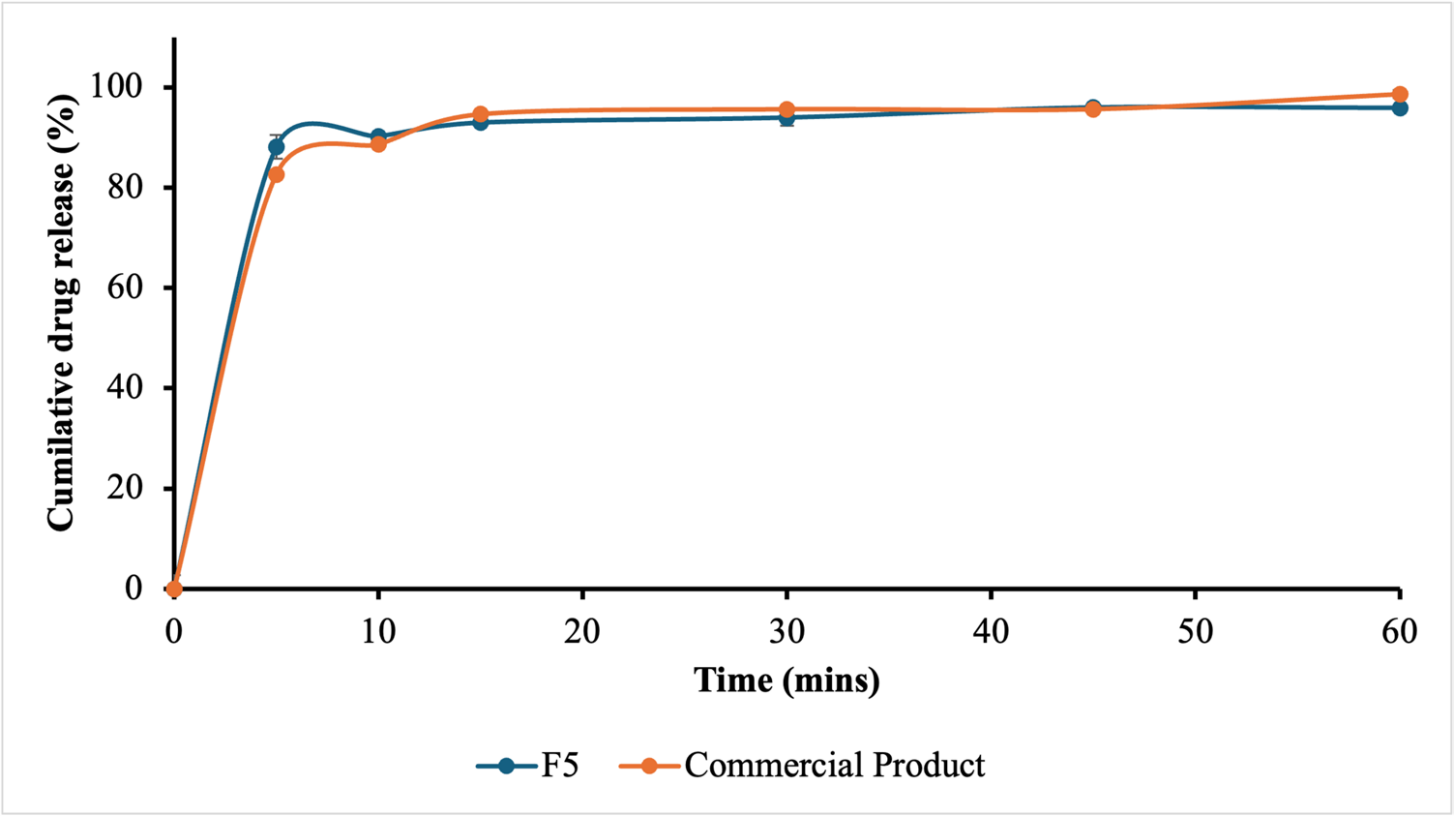
Dissolution profile of F5(l/S: 0.52) and commercial product in 0.1 N HCl.

0.1 N HCl was selected as the dissolution medium due to its biological relevance, and the test was conducted with 500 mL, which is sufficient to dissolve the 460.55 mg of QTF (Eq. 400 mg base) present in the unit dose, given QTF’s high solubility in acidic conditions (~ pH 1.0), which is approximately ranging from 35.8 to 94.3 mg/mL, reported in various studies [45, 46]. The F5 formulation released 95% of QTF within 5 min, as shown in Fig. 6. Two factors contributed to this rapid dissolution: first, QTF's high solubility in 0.1 N HCl, and second, the presence of porous channels within the granules despite the high shear applied during processing.

The comparison between F5 and the commercially marketed product's dissolution behaviour showed that both had similar dissolution release rates and profiles, with more than 85% release within 15 min. This indicates that F5 is comparable to the commercially marketed product.

## Conclusion

The TSWG platform has been widely used in several pharmaceutical industries due to its robustness, scalability, and most importantly, to preserve the crystallinity of the API. This novel study aimed to evaluate how each process parameter affects granule quality and the development of QTF immediate release tablets. We established a clear relationship between various parameters. The optimum L/S ratio varied with the material properties (binder used) as well as the screw configuration used. Overall, an increase in L/S showed an improvement in flow properties, as well as the production of more uniform-sized granules with a lower percentage of fines. Increasing the number of mixing zones inside the barrel creates higher shear, resulting in optimal granulation while using less liquid. This potentially reduces the drying time of granules. The tablets that were compressed with granules processed with three mixing zones and 0.52 L/S ratio surprisingly disintegrated and released ~ 95% of QTF rapidly within 5 min, attributing to the drug’s higher solubility in 0.1 N HCl and the possible presence of porosity of granules. Overall, these results emphasize the potential of continuous wet granulation as an alternative approach to enhance pharmaceutical manufacturing processes.

## Data Availability

All data generated or analysed during the study are included in this article.
